# A potential inhibitory function of draxin in regulating mouse trunk neural crest migration

**DOI:** 10.1007/s11626-016-0079-0

**Published:** 2016-09-20

**Authors:** Sanbing Zhang, Yuhong Su, Jinbao Gao, Chenbing Zhang, Hideaki Tanaka

**Affiliations:** 1 Department of Hand and Foot Surgery, The Third Hospital of Shijiazhuang City, 15 South Tiyu Street, Shijiazhuang, 050000 People’s Republic of China; 2 Department of Human Anatomy, Hebei Medical University, No. 361 East Zhongshan Road, Shijiazhuang, Hebei Province 050017 People’s Republic of China; 3 Division of Developmental Neurobiology, Graduate School of Medical Sciences, Kumamoto University, Kumamoto, Japan

**Keywords:** Draxin, Axon guidance, Trunk neural crest, Migration, Actin cytoskeleton, Spinal cord

## Abstract

Draxin is a repulsive axon guidance protein that plays important roles in the formation of three commissures in the central nervous system and dorsal interneuron 3 (dI3) in the chick spinal cord. In the present study, we report the expression pattern of mouse draxin in the embryonic mouse trunk spinal cord. In the presence of draxin, the longest net migration length of a migrating mouse trunk neural crest cell was significantly reduced. In addition, the relative number of apolar neural crest cells increased as the draxin treatment time increased. Draxin caused actin cytoskeleton rearrangement in the migrating trunk neural crest cells. Our data suggest that draxin may regulate mouse trunk neural crest cell migration by the rearrangement of cell actin cytoskeleton and by reducing the polarization activity of these cells subsequently.

## Introduction

Neural crest cells arise in the developing neural tube. The neural crest cells can be subdivided into four distinct major axial populations: cranial, cardiac, vagal, and trunk. Each population of cells migrates along unique pathways and contributes to specific cell and tissue types that are specific to their axial level of origin. In the trunk region of most animals, neural crest cells travel in two pathways, a medial pathway through the somitic mesoderm or between the neural tube and somites and a dorsolateral pathway between the somites and the overlying ectoderm (Morin-Kensicki and Eisen 1997). There are, however, species-specific differences in neural crest cell formation and migration between the mouse and avian species (Nichols 1986; Serbedzija et al. 1992). Murine neural crest cell formation and migration commence at approximately four to five somite stages in the caudal midbrain and rostral hindbrain regions and simultaneously proceed rostrally towards the forebrain and caudally towards the tail in a wave-like pattern (Trainor 2005). In avian embryos, neural crest cell migration commences after neural tube closure. In the mouse embryo, however, neural crest cell formation and migration begin well before the bilateral halves of the neural plate have fused (Peeters et al. 1998). Several axon guidance molecules are involved in mediating neural crest cell migration. Semaphorins, especially class 3 semaphorins, have been shown to play important roles in regulating neural crest cell migration (Eickholt et al. 1999; Osborne et al. 2005). The Slit/Robo interaction prevents neural crest cells from migrating along the dorsolateral pathway (Jia et al. 2005). Eph family receptors and their ephrin ligands play a role in the interactions between the neural crest and sclerotomal cells (Santiago and Erickson 2002; Kasemeier-Kulesa et al. 2006).

Dorsal repulsive axon guidance protein, or draxin, is a secreted repulsive axon guidance protein (Islam et al. 2009). Draxin was identified in a signal sequence trap screening using a motoneuron, floor plate, and roof plate complementary DNA (cDNA) library derived from chick embryos. Draxin does not share homology with any known guidance cues, and it has been shown to be required for the development of the spinal cord and forebrain commissures (Islam et al. 2009). Draxin also plays an important role during hippocampus, lateral olfactory tract, and olfactory bulb development in mice and during dorsal interneuron 3 (dI3) development in the chick spinal cord (Ahmed et al. 2010; Su et al. 2010; Zhang et al. 2010). The draxin protein localizes to the migration pathways of the chick trunk neural crest cells and inhibits chick neural crest cell migration in vitro. The overexpression of draxin causes some early-born chick neural crest cells to change their migration pathway to dorsolateral pathway (Su et al. 2009).

While it has been well established that draxin plays an important role during chick neural crest cell migration, its function during mouse neural crest cell migration and the functional mechanism remain to be elucidated. In the present study, we report the draxin expression pattern in the mouse spinal cord. Additionally, we show that the longest net migration length of a migrating mouse trunk neural crest cell was significantly reduced in the presence of draxin and the migrating neural crest cells avoided the purified mouse draxin fusion protein-coated region. Furthermore, the relative number of apolar mouse neural crest cells increased in the presence of chick draxin. Draxin caused actin cytoskeleton rearrangement in the migrating trunk neural crest cells in vitro. No significant difference of trunk neural crest cell migration could be detected between wild-type and draxin knockout mice. Our data suggest that draxin may regulate mouse trunk neural crest cell migration by the rearrangement of cell actin cytoskeleton and by reducing the polarization activity of these cells subsequently in vitro.

## Materials and Methods

### Chick embryos

Fertilized White Leghorn chick eggs were obtained from local commercial sources. All the eggs were incubated at 38°C until the embryos reached the desired Hamburger and Hamilton (HH) stages (Hamburger and Hamilton 1951).

### Mouse embryos

All of the experiments were conducted in accordance with the guidelines for the care and use of animals approved by the Animal Care and Use Committee of Hebei Medical University. All efforts were made to minimize the number of animals used and their suffering. The wild-type (WT) mice used in this study were obtained from a local company. Draxin heterozygous knockout mice and draxin homozygous knockout mice were obtained from a colony at the animal center at Kumamoto University (draxin knockout/β-galactosidase (β-gal) knockin mice) (Islam et al. 2009).

### Neural tube explant culture and stripe assay

Embryonic day 9.5 (E9.5) WT mouse embryos were used for explant culture. Newly formed one to ten somite level neural tubes were collected and cultured on fibronectin-coated dishes, as previously described (Gammill et al. 2006; Su et al. 2009). Draxin cDNA tagged with myc and His was cloned into the pMES-internal ribozyme entry site-enhanced green fluorescent protein (pMES-IRES-EGFP) vector. COS7 cells were transfected with draxin (pMES-myc-His-draxin-IRES-EGFP) *cDNA* plasmid or control vector pMES-IRES-EGFP plasmid to produce the desired conditioned medium (CM), as previously described (Islam et al. 2009; Ahmed et al. 2010; Su et al. 2009, 2010). Four days after transfection, the supernatant was harvested and used as CM. The neural tubes were cultured for 18 h in the draxin CM or control CM, both of which were diluted in DMEM-F12/10% fetal bovine serum (FBS) (1:1).

For stripe assay, the myc-His-mouse draxin vector was stably introduced into 293 cells and expanded in a serum-free culture medium. Then, the draxin protein was purified by Ni-NTA beads (Islam et al. 2009). The preparation of alternately coated dishes was performed as previously described (Wang and Anderson 1997; Knöll et al. 2007). The plastic matrix which contains a channel system was attached to plastic Petri dishes, and 100 μl medium was used to fill the channel system. After 1 h of incubation, the matrix was removed and then the plastic matrix-covered region was coated with fibronectin before neural tube culture. Purified human IgG was used as control. The concentration was 5 μg/ml for control medium and 500 μg/ml for purified draxin fusion protein.

### In situ hybridization

To evaluate the *draxin* expression pattern, in situ hybridization experiments were conducted using digoxigenin (DIG)-labeled RNA antisense probes, as previously described (Schaeren-Wiemers and Gerfin-Moser 1993; Okafuji and Tanaka 2005). HH-stage 14–15 whole-mount chick embryos and transverse sections of E10.5 mouse spinal cord were used in this analysis.

### Immunohistochemistry, β-gal staining, and scanning electron microscopy

The samples were fixed in 4% PFA for 30 min for explants or 1 h for transverse sections and stained with the primary antibodies (anti-p75 antibody, Bioworld, Irving, TX; anti-mouse draxin monoclonal antibody, a gift from Prof. Tanaka; anti-cortactin antibody, Abcam, Cambridge, UK), as previously described (Gammill et al. 2006; Islam et al. 2009; Su et al. 2009; Ahmed et al. 2011). Briefly, the samples were blocked in 5% skim milk in PBS at RT for 1 h after washing in PBST (0.3% Triton X-100 in PBS). Next, the samples were incubated in primary antibodies diluted in blocking buffer at 4°C overnight. Cy3-conjugated secondary antibodies were diluted 1:300. For actin stress fiber staining, the fixed neural crest cells in cultured explants were incubated with Alexa 488-conjugated phalloidin (Cell Signaling, Danvers, MA) for 30 min before observation. For the cultured explant experiment, the average net migration lengths of the neural crest cells from the neural tube were measured and compared. The largest vertical dimension between a single migrating neural crest cell at the leading edge and the neural tube explants was recorded as the longest net migration path. β-gal staining was conducted as previously described (Nagy et al. 2003). For scanning electron microscopy, cultured mouse trunk neural crest cells were prepared following a standard procedure and observed using a Hitachi S-3500N scanning electron microscope.

## Results

### The mouse draxin expression pattern is similar, but not the same, to the chick expression pattern in the spinal cord

**Figure 1.**
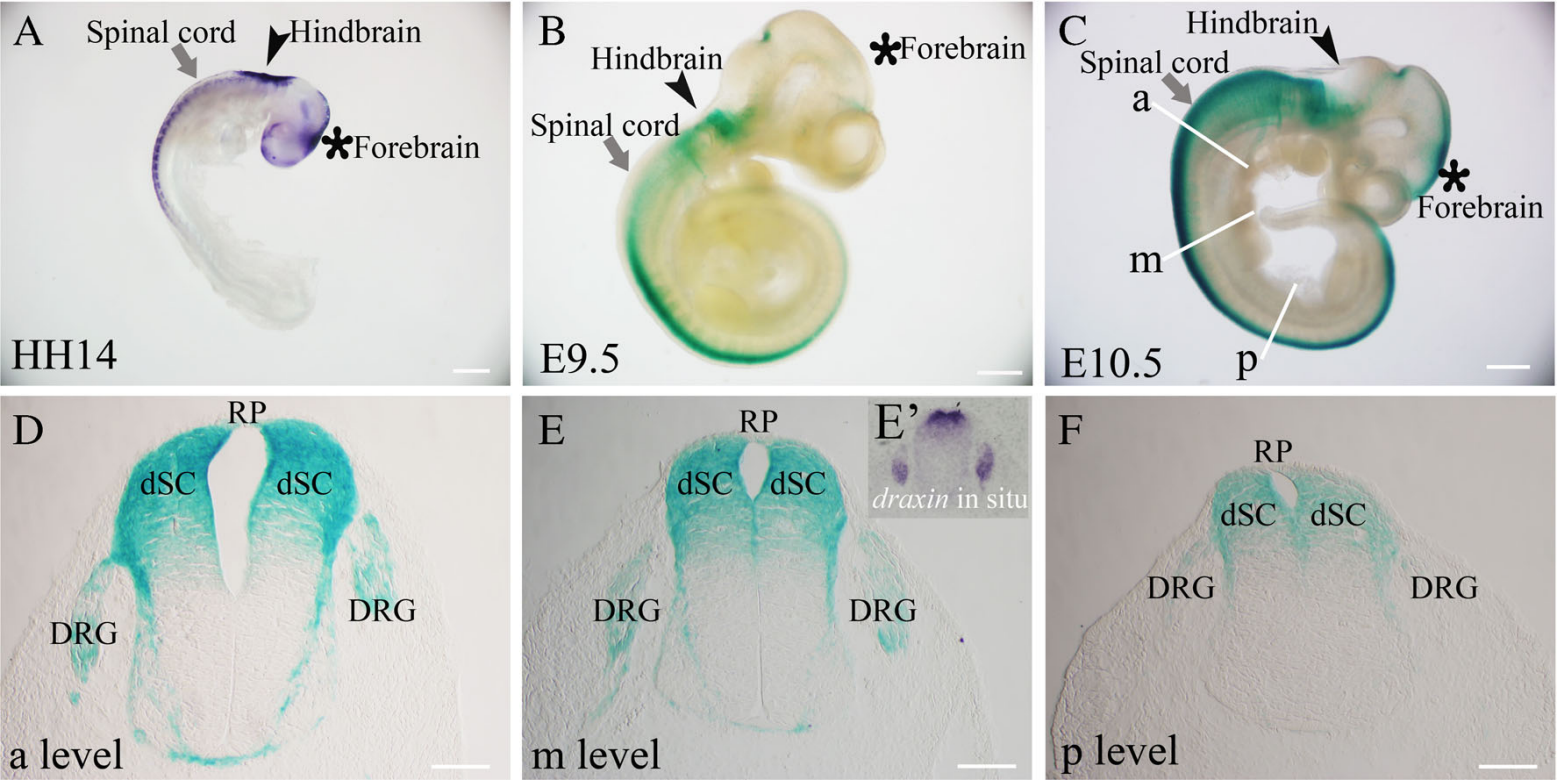
The comparison of draxin expression pattern between mouse and chick. (*A*) Whole-mount *draxin* in situ hybridization of an HH stage 14 chick embryo. (*B*) Whole-mount β-gal staining of an E9.5 mouse embryo. (*C*) Whole-mount β-gal staining of an E10.5 mouse embryo. (*D*) β-gal staining of a transverse section of the mouse embryonic spinal cord in the anterior region. (*E*) β-gal staining of a transverse section of the mouse embryonic spinal cord in the middle region. (*E’*) *Draxin* in situ hybridization of a transverse section of the E10.5 mouse embryonic spinal cord in the middle region. (*F*) β-gal staining of a transverse section of the mouse embryonic spinal cord in the posterior region. The *arrows* indicate the spinal cord. The *arrowheads* indicate the hindbrain. The *asterisks* indicate the forebrain. *RP* roof plate, *dSC* dorsal spinal cord, *DRG* dorsal root ganglion. *Scale bars* = 500 μm (*A*–*C*) and 100 μm (*D*–*F*).

A null *draxin* mutation was produced by replacing the second exon, which contains the ATG start codon, with a lacZ-neo selection cassette. Because the β-gal staining results were comparable to the *draxin* in situ hybridization results (Islam et al. 2009; Zhang et al. 2010), we used β-gal staining on draxin heterozygous mice to evaluate the expression pattern of mouse draxin. To compare the expression patterns of chick and mouse draxin, we selected the developmental stage at which trunk neural crest cells begin to migrate out from the neural tube. In chick, this stage is Hamburger and Hamilton (HH) stages 14 and 15, and in mice, this stage is E9.5. The stages and the main structures in the trunk spinal cord are similar in early-developing embryonic chick and mouse (Hamburger and Hamilton 1951; Krull 2001; Dupin and Le Douarin 2003; Islam et al. 2009). In the HH stage 14–15 chick embryos, chick *draxin* is expressed in the forebrain, hindbrain, and anterior regions of the spinal cord (Fig. 1 *A*). In the E9.5 mouse embryo, mouse *draxin* is expressed in the forebrain, hindbrain, and most regions of the spinal cord (Fig. 1 *B*). Chick *draxin* expression has a distinct cranial-to-caudal gradient, which was reported in our previous studies (Su et al. 2009; Ahmed et al. 2010). In the E10.5 mouse embryo, *draxin* mRNA was expressed in the forebrain, hindbrain, and most of the spinal cord, and the expression appears to be more intense than the expression pattern observed in the E9.5 mouse embryo (Fig. 1 *C*).

The mouse *draxin* expression pattern also appears to have a cranial-to-caudal gradient, especially in the spinal cord, and β-gal staining of transverse spinal cord sections confirmed this expression pattern gradient. In the anterior region of the mouse spinal cord, draxin appeared to be strongly expressed in the roof plate, dorsal spinal cord, and some portions of the dorsal root ganglion (DRG). Draxin also appeared to be expressed in some portions of the commissures and basement membrane (Fig. 1 *D*). Draxin appeared to be expressed in the same regions in the middle region of the mouse spinal cord, but the intensity level was weaker than the intensity level observed in the anterior region (Fig. 1 *E*). In situ hybridization of transverse sections (Fig. 1 *E’*) demonstrated that β-gal staining was comparable to the pattern observed using *draxin* in situ hybridization. The intensity of expression in the posterior region of the mouse spinal cord was even weaker compared with that in the anterior and middle regions. Only weak draxin expression was detected in the roof plate, dorsal spinal cord, and some portions of the DRG in the posterior region of the mouse spinal cord (Fig. 1 *F*). At early developmental stage, chick draxin is detected in the dermomyotome, but not in the DRG (Su et al. 2009). But, mouse draxin could be detected in the DRG. There were some differences in the draxin expression pattern between chick and mouse in the brain. There appeared to be strong chick *draxin* expression in the forebrain and dorsal hindbrain regions of HH stage 14–15 chick embryos, but the expression of mouse *draxin* in the E9.5 mouse embryonic forebrain and dorsal hindbrain regions appeared to be weak. *Draxin* expression, however, did appear to be much stronger in the E10.5 mouse embryonic forebrain and hindbrain regions.

### Draxin inhibits mouse neural crest cell migration from neural tube explants in vitro

**Figure 2.**
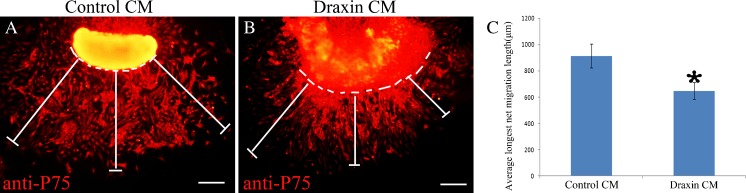
Mouse draxin inhibits mouse trunk neural crest cell migration in vitro. The *bars around each explant* show three different net migration paths taken by neural crest cells. This value was defined as the largest vertical dimension between a single migrating neural crest cell at the leading edge and the neural tube explant. E9.5 mouse neural tubes were cultured in control CM (*A*) or mouse draxin CM (*B*) and then stained with an anti-p75 antibody. (*C*) The *column diagram* shows that the average longest net migration length was significantly shorter in the presence of draxin. The *dashed line* indicates the edge of the neural tube. *P < 0.001. *Scale bars* = 100 μm.

To determine the function of draxin during mouse trunk neural crest cell migration, we conducted an in vitro trunk neural tube explant culture experiment. After anti-p75 immunostaining, the mouse neural crest cells were observed in a halo pattern around the neural tube. We also determined the percentage of migrating neural crest cells which were anti-p75 positive. A total of 18 explants cultured with the control culture medium were used for counting. One visual field from the leading edge portion was selected, and the ratio of anti-p75-positive cell to total cell was counted. The average percentage of anti-p75-positive cell was about 95.8%. The largest vertical dimension between a single migrating neural crest cell at the leading edge and the neural tube was measured and recorded as the longest net migration length. The longest net migration lengths measured in three different directions were recorded for each explant. The middle direction was vertical downward. The angle between the other two directions with the middle one was about 45 ° in the left and right sides, respectively. We calculated the mean of these three longest net migration lengths and used this value as the average net migration length for each explant in our comparative analyses. A total of 24 explants cultured in the control conditioned medium (CM) and 22 explants cultured in the mouse draxin CM from three independent experiments were used for the statistical analyses. The standard *t* test was used to determine statistically significant differences. Compared with control explants, the average longest net migration length was significantly reduced in the presence of mouse draxin (Fig. 2).

### Draxin inhibits mouse neural crest cell migration in vitro by reducing cell polarization

**Figure 3.**
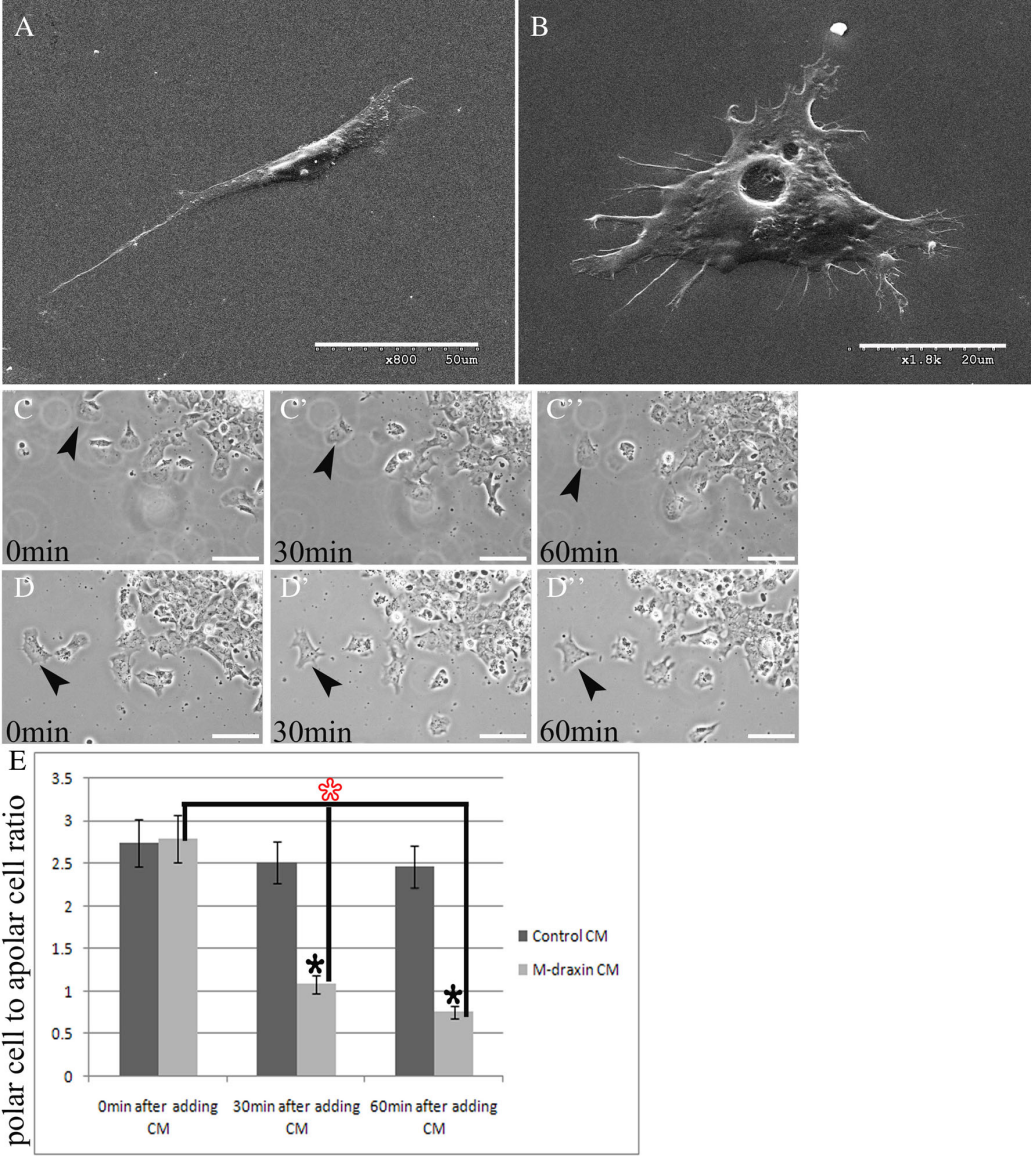
The apolar mouse neural crest cell number increases in the presence of draxin. (*A*) A representative polar cell shape of a migrating mouse neural crest cell. (*B*) A representative apolar cell shape of a migrating mouse neural crest cell. (*C*) Zero min after adding the control CM. (*C’*) Thirty min after adding the control CM in the same view as shown in (*C*). (*C”*) Sixty min after adding the control CM in the same view as shown in (*C*). (*D*) Zero min after adding the draxin CM. (*D’*) Thirty min after adding the draxin CM in the same view as shown in (*D*). (*D”*) Sixty min after adding the draxin CM in the same field as shown in (*D*). (*E*) The *column diagram* shows that the ratio of polar mouse neural crest cells to apolar mouse neural crest cells decreases in the presence of mouse draxin. There was a larger decrease in the ratio for the 60-min treatment group compared with the 30-min treatment group. The *arrows* indicate the same cells under different treatments, respectively. The *black asterisks* indicate the comparison of the draxin treatment group with the control group. The *red asterisk* indicates the comparison of the 30- or 60-min draxin treatment group with the 0-min draxin treatment group. *P < 0.001. *Scale bars* = 50 μm in (*A*), (*E*); 20 μm in (*B*).

To confirm the previous result and investigate the inhibitory effect of mouse draxin on neural crest cell migration, we counted the number of cell shape changes at different treatment time intervals. To reduce the influence of contact inhibition on our results, only single cells at the leading edge which were rarely in contact with each other were analyzed. We used scanning electron microscopy to observe and illustrate the typical cell shape types. Cells with leading protrusions, such as one large lamellipodium and/or one filopodium or two small lamellipodia and/or two small filopodia, at the front and a retracting protrusion at the back were included as polar cells (Fig. 3 *A* shows the cell with two small lamellipodia). Cells without leading protrusions at the front and a retracting protrusion at the back, or very small lamellipodia and/or filopodia in different directions, were included as apolar cells. The typical apolar cell had many protrusions that were dispersed in different directions (Fig. 3 *B* shows the cell with very small filopodia in different directions).

We counted the cell shape changes at three time points, 0, 30, and 60 min, after adding the CM. The representative cell shape changes under different treatment times are shown in Fig. 3 *C*, *D”*. All the migrating cells from one explant were captured under ×200 magnification. All the single cells at the leading edge that were rarely in contact with each other in each ×200 magnification field were counted. We calculated the ratio of polar cells to apolar cells in each field and then averaged the ratios from all the fields from one explant. This average ratio of polar cells to apolar cells was used for statistical analysis. At least 18 explants from three independent experiments for each group were used. We lastly calculated the average ratio of polar cells to apolar cells from all the explants in each group and performed a standard *t* test. A high ratio indicates that the polar cell number is relatively higher, and a low ratio indicates that the apolar cell number is relatively higher. For the cell treated with the control CM, the same cell was counted as polar at 0, 30, and 60 min of treatment (Fig. 3 *C*, *C”*). For the same cell treated with the draxin CM, the cell shape was counted as polar at 0 min and apolar at 30 and 60 min of treatment (Fig. 3 *D’*, *D”*). There was no significant difference in the ratio of polar cells to apolar cells calculated for the 0-min time point (i.e., immediately after adding the mouse draxin CM) compared with the control CM (Fig. 3 *E*). The ratio of polar cells to apolar cells was significantly reduced at 30 and 60 min after adding the mouse draxin CM compared with the control CM (Fig. 3 *E*).

These results indicate that the relative number of apolar cells increased in the presence of mouse draxin. When comparing the mouse draxin CM treatment time intervals, the ratio of polar cells to apolar cells was reduced in the 30- and 60-min treatment groups compared with the 0-min treatment groups (Fig. 3 *E*). Furthermore, the ratio was smaller for the 60-min treatment group compared with the 30-min treatment group (Fig. 3 *E*). This result suggests that the relative number of apolar cells increases with treatment time.

### Purified mouse draxin fusion protein inhibits neural crest cell migration in a stripe assay

**Figure 4.**
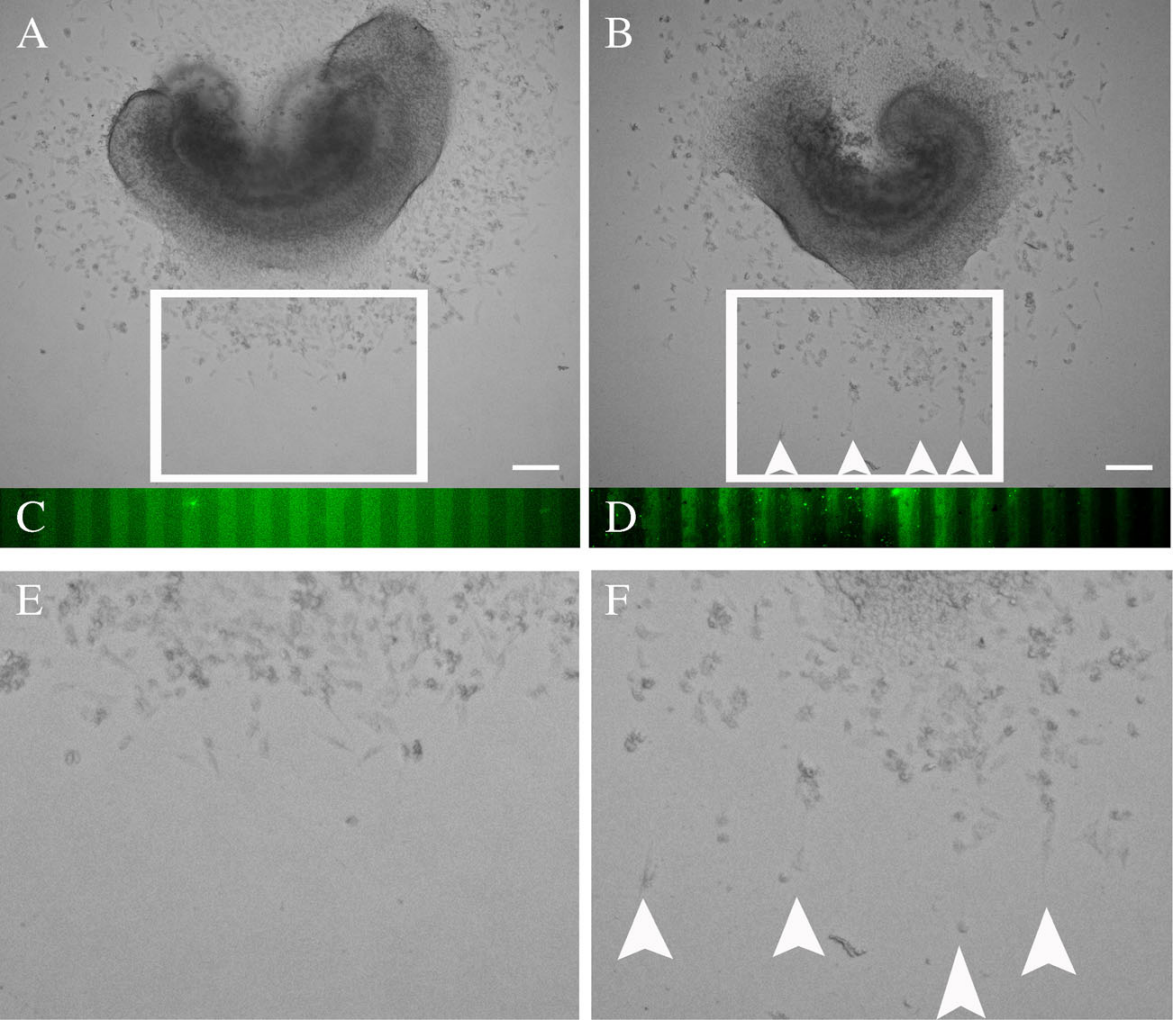
Purified mouse draxin fusion protein inhibits neural crest cell migration in a stripe assay. (*A*) Neural crest cell migration in control human IgG alternately coated stripes. (*B*) Neural crest cell migration in purified mouse draxin fusion protein alternately coated stripes. (*C*) Anti-human IgG fluorescence staining shows the alternately coated human IgG. (*D*) Anti-draxin fluorescence staining shows the alternately coated purified mouse draxin fusion protein. (*E*) Higher magnification of the *boxed* region in (*A*). (*F*) Higher magnification of the *boxed* region in (*B*). *Arrowheads* in (*B*), (*F*) show the biased migration of neural crest cells migrate towards the draxin-free stripes. *Scale bars* = 100 μm.

To exclude the possibility of indirect or nonspecific effects caused by the draxin CM, we performed a stripe assay using the purified mouse draxin fusion protein. The neural crest cells migrated evenly around the neural tube which was cultured on the human IgG alternately coated dish (Fig. 4 *A*, *E*). The alternately coated human IgG was displayed by anti-human IgG immunostaining (Fig. 4 *C*). In the leading edge, the neural crest cells migrated unevenly around the neural tube which was cultured on the purified mouse draxin fusion protein alternately coated dish (Fig. 4 *B*, *F*). The alternately coated purified draxin protein was displayed by anti-draxin immunostaining (Fig. 4 *D*). The migrating neural crest cells in the leading edge were inclined to avoid the purified mouse draxin-coated stripes (arrowheads in Fig. 4 *B*, *F*).

### Draxin changes the organization of the actin cytoskeleton in vitro

**Figure 5.**
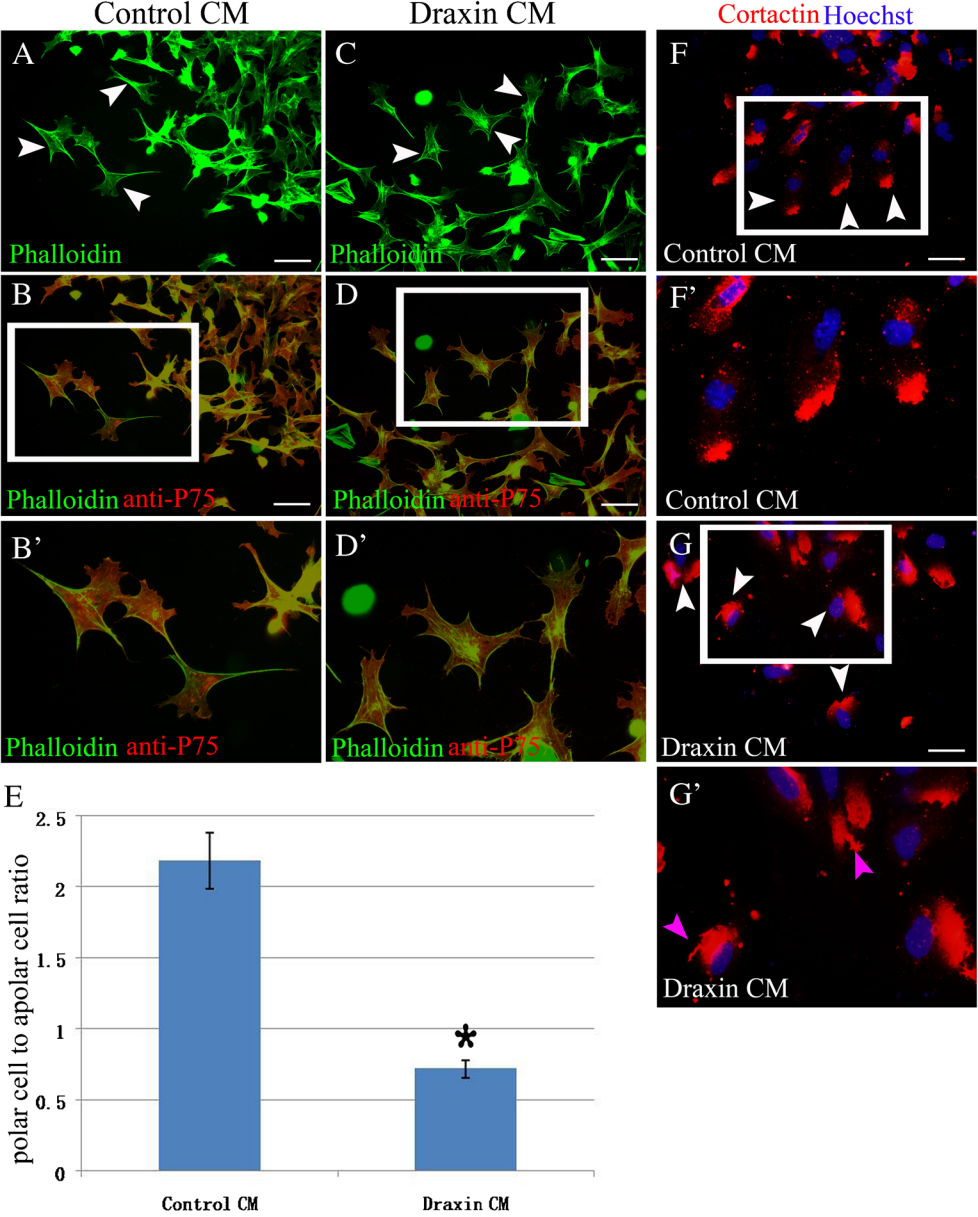
The actin cytoskeleton arrangement changes in the presence of draxin. (*A*) A representative arrangement of actin cytoskeleton by phalloidin staining in the presence of control CM. Two major stress fiber bundles align to the long axis of the cell body (*arrowheads*). (*B*) The anti-P75 and phalloidin double staining in the same view with (*A*). (*B’*) Higher magnification of the *boxed* region in (*B*). (*C*) A representative arrangement of actin cytoskeleton by phalloidin staining in the presence of draxin CM. Major stress fiber bundles encircle the cell cortex in a polygonal arrangement (*arrowheads*). (*D*) The anti-P75 and phalloidin double staining in the same view with (*C*). (*D’*) Higher magnification of the *boxed* region in (*D*). (*E*) The *column diagram* shows that the ratio of polar cells to apolar cells was significantly reduced in the presence of draxin. (*F*) A representative arrangement of actin cytoskeleton by cortactin staining in the presence of control CM. Hoechst staining shows the cell nuclear. Cortactin-positive signal locates near one pole of the cell body and far away from cell nuclear (*arrowheads*). (*F’*) Higher magnification of the *boxed* region in (*F*). (*G*) A representative arrangement of actin cytoskeleton by cortactin staining in the presence of draxin CM. Hoechst staining shows the cell nuclear. Cortactin-positive signal locates in one side of the cell body dispersedly and close to cell nuclear (*arrowheads*). (*G’*) Higher magnification of the *boxed* region in (*G*). Some long irregular projections protrude out from the cell surface (*pink arrowheads*). *P < 0.05. *Scale bars* = 50 μm.

As the cell protrusive activity and the formation of focal contacts are dynamically regulated by the actin cytoskeleton, we used Alexa 488-conjugated phalloidin staining to examine actin cytoskeletal organization in vitro. The explants were cultured for 60 min and fixed for immunostaining. The representative cell actin cytoskeleton structure changes are shown in Fig. 5. In the mouse neural crest cells that were cultured with the control CM, the two major stress fiber bundles align to the long axis of the cell body in most of the cells (Fig. 5 *A*). This was one typical polar cell structure. In the mouse neural crest cells that were cultured with the draxin CM, the major stress fiber bundles encircle the cell cortex in a polygonal arrangement in most of the cells (Fig. 5 *C*). This was one typical apolar cell structure. Single cells at the leading edge that were rarely in contact with each other were used in this comparison. The cells used for analyses were also anti-P75 positive (Fig. 5 *B*, *B’*, *D*, *D’*). We calculated the number of polar cells and apolar cells in each field of one explant and then got the total number for this explant. The ratio of polar cells to apolar cells was counted for this explant. At least 16 explants from three independent experiments for each group were used. We lastly calculated the average ratio of polar cells to apolar cells from all the explants in each group and performed a standard *t* test. The ratio of polar cells to apolar cells was significantly reduced in the presence of the draxin CM compared with the control CM (Fig. 5 *E*).

Cortactin is one regulator of F-actin and important for regulating branched actin assembly. It could be detected in the cell cortex or perinuclear region (Weed and Parsons 2001; Kirkbride et al. 2011; Hajdu et al. 2015). To further display the change of actin cytoskeleton, we performed cortactin and Hoechst immunostaining after neural tube explant culture. The distribution of cortactin in cells migrating in the leading edge was checked. In the presence of control CM, most cells showed a polar distribution of cortactin-positive signal. Assembled cortactin located near one pole of the cell body and far away from cell nuclear (Fig. 5 *F*, *F’*). In the presence of draxin CM, most cells lost the typical polar distribution of cortactin-positive signal. Cortactin located in one side of the cell body dispersedly and very close to cell nuclear (Fig. 5 *G*, *G’*). Some long irregular projections protruded out from the cell surface (pink arrowheads in Fig. 5 *G’*). The cells in the leading edge were selected for counting. We calculated the number of polar cells and apolar cells for one explant. The ratio of polar cells to apolar cells was counted for this explant. At least 18 explants from three independent experiments for each group were used. We finally calculated the average ratio of polar cells to apolar cells from all the explants in each group and performed a standard *t* test. The ratio of polar cells to apolar cells was 0.47 ± 0.03 (mean ± SD) in the presence of draxin CM. The ratio of polar cells to apolar cells was 2.65 ± 0.17 (mean ± SD) in the presence of control CM. The ratio of polar cells to apolar cells was significantly reduced in the presence of draxin CM compared with control CM (*P* < 0.05).

### No significant difference of trunk neural crest cell migration could be detected between wild-type and draxin knockout mice

**Figure 6.**
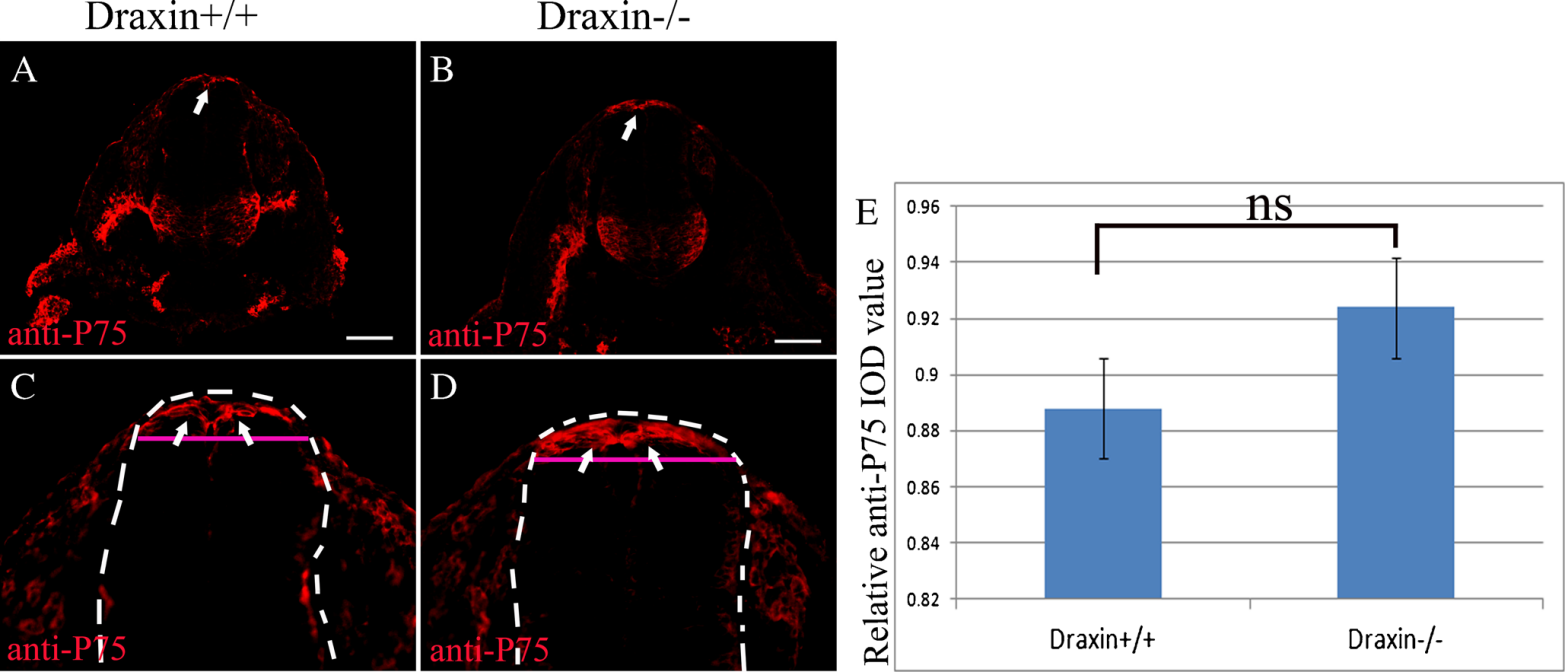
No significant difference of anti-p75-positive neural crest cells could be detected in draxin knockout mice. Transverse sections from the E10.5 mouse embryonic spinal cord were used. (*A*) Anti-p75 immunostaining of wild-type (draxin+/+) mouse. (*B*) Anti-p75 immunostaining of draxin knockout (draxin−/−) mouse. (*C*) Higher magnification of the dorsal portion of the spinal cord in (*A*). (*D*) Higher magnification of dorsal portion of the spinal cord in (*B*). The *arrows* indicate the p75-positive cells near the roof plate. The *white outline* shows the region for total IOD value measurement. The *IOD value above the pink straight line inside the white outline* shows the region for the IOD value near the roof plate region measurement. The relative anti-p75 IOD value is higher in the draxin−/− mice compared with draxin+/+ mice. But, no significant difference could be found between draxin−/− and draxin+/+ mice. *Scale bars* = 100 μm.

To further reveal the function of draxin, we checked the trunk neural crest cell migration using E10.5 wild-type (Fig. 6 *A*, *C*) and draxin knockout (Fig. 6 *B*, *D*) mice embryos. The transverse section from the thoracic trunk spinal cord region was used, and six pairs of wild-type and draxin knockout embryos (littermates) were used for anti-p75 immunostaining. At least five sections per embryo were counted. Draxin might be involved in the restriction of chick neural crest cell migration pathway by inhibiting them from invading a draxin-expressing region (Su et al. 2009). We speculated that mouse draxin expressed in the roof plate and dorsal spinal cord might have some repulsive function for neural crest cells migrating out from the spinal cord. For this reason, we measured the integrated optical density (IOD) value of anti-p75 immunostaining near the roof plate region. To reduce the individual difference between sections, we used a relative anti-p75 IOD value. The IOD value inside the whole area of the spinal cord was counted as the total IOD value. Then, we made one straight horizontal line from the lowest border of anti-p75-positive region. The IOD value above this line inside the whole area of the spinal cord was counted as the IOD value near the roof plate region. Finally, we got the relative anti-p75 IOD value (IOD value near the roof plate region/total IOD value). We calculated the mean and SD for comparison (Fig. 6 *E*). Although we observed a small increase of the relative anti-p75 IOD value in draxin knockout mice (Fig. 6 *B*, *D*) compared to wild-type mice (Fig. 6 *A*, *C*), this difference was not statistically significant (Fig. 6 *E*).

## Discussion

Neural crest cell migration is a complex process that involves interactions between migrating cells and cues from the local environment. Draxin is required for the development of the spinal cord and forebrain commissures (Islam et al. 2009). Draxin is also involved in the migration of dI3 interneurons in the chick spinal cord (Su et al. 2010). In the chick spinal cord, draxin is expressed in the roof plate, the dorsal lip of the dermomyotome, and the lateral region of the spinal cord adjacent to the dorsal basement membrane; these regions are near migrating chick neural crest cells. Draxin reduces the ability of chick neural crest cells to polarize in vitro (Su et al. 2009). However, what is the function of draxin during mouse embryonic neural crest cell migration? In this study, we first evaluated the mouse draxin expression pattern at the stages at which neural crest cells migrate out from the neural tube. Our results showed that the expression patterns of mouse and chick draxin in the spinal cord are similar, but not the same (Fig. 1). These similar expression patterns indicate that mouse and chick draxin may have a similar function during neural crest cell migration.

To investigate this possibility, we performed in vitro mouse neural tube culture experiments. Compared with control explants, the average longest net migration length was significantly reduced in the presence of mouse draxin (Fig. 2). In addition, the relative number of apolar neural crest cells was higher following different times of draxin CM treatment. Furthermore, the relative number of apolar neural crest cells increased as the time interval of draxin treatment increased (Fig. 3). This result indicates that mouse draxin may have inhibitive effects during neural crest cell migration. By stripe assay, we found that the migrating neural crest cells were avoiding the purified draxin protein-coated region (Fig. 4). This result further reveals the inhibitive function of draxin during neural crest cell migration. On the other hand, the expression pattern and intensity are not the same between chick and mouse, especially in the brain region. This faint difference indicates that there might be some different functions or function time might be different between chick and mouse. We plan to analyze the detailed difference in our future studies.

Neural crest cells constitute a multipotent population of migratory cells that arise in the dorsal neural tube. These cells then undergo an epithelial-to-mesenchymal transition (EMT), delaminate, and then migrate (Barembaum and Bronner-Fraser 2005; Noden and Trainor 2005; Sauka-Spengler and Bronner-Fraser 2006; Duband 2010). Gene expression and morphological changes in neural crest cell precursors facilitate the EMT, which is characterized by a loss of the cell’s adherent, epithelial nature, and the subsequent acquisition of a mesenchymal, motile phenotype that exemplifies the migratory neural crest cells (Taneyhill et al. 2007; Thiery et al. 2009; Lim and Thiery 2012). Actin-based migratory cellular protrusions, such as lamellipodia, are required for directed cell migration, and these protrusions may inhibit cell migration if there is a defect in cell polarization. Given that the function of draxin reduced cell polarity, we examined actin cytoskeleton changes in vitro (Fig. 5). In the presence of draxin, the major stress fiber bundles encircle the cell cortex in a polygonal arrangement, not parallel to the long axis of the cell body. Cortactin is one regulator of F-actin and important for regulating dynamically branched actin assembly. Dynamically branched actin assembly is critical for other aspects of cell motility, including formation of protrusive motility structures and membrane trafficking to promote directional cell motility (Weed and Parsons 2001; Kirkbride et al. 2011; Hajdu et al. 2015). Draxin caused the change of cortical actin assembly character by anti-cortactin immunostaining (Fig. 5). This result indicated that draxin inhibits the formation of protrusive motility structures and membrane trafficking. This result strongly indicated that draxin inhibits mouse neural crest cell migration by rearranging the actin cytoskeleton. Actin stress fibers play important roles in the retraction of the trailing edge of the cell and contraction of the cell body (Etienne-Manneville 2004). Based on all the above results, we can speculate that draxin caused rearrangement of the actin cytoskeleton. As a result, the polarized distribution of actin cytoskeleton was lost. This result further caused that the formation of protrusions, the retraction of the trailing edge of the cell, and contraction of the cell body were inhibited. Lastly, these effects resulted in a reduction of cell polarization activity and the inhibition of cell migration.

No significant difference of trunk neural crest cell migration could be detected between wild-type and draxin knockout mice. The development of mouse embryos was much complex compared with chick embryos. There are many inhibitory factors, which had similar function with draxin during regulating neural crest cell migration, located inside the embryonic spinal cord. The redundant function of these inhibitory factors might be one of the reasons that there was no significant difference could be detected between wild-type and draxin knockout mice. All the above results will give an indication for further checking the functional difference between different species and detailed signaling pathway during regulating neural crest cell migration of draxin.

## Conclusions

During early development stage, draxin was strongly expressed in the roof plate, dorsal spinal cord, and some portions of the dorsal root ganglion. These regions were along the migration pathway of early migrating neural crest cells. The in vitro explant culture experiments showed that the migration of trunk neural crest cells was inhibited by draxin protein. In the presence of mouse draxin, the cell polarization activity was decreased and this decrease might be caused by the rearrangement of cell actin cytoskeleton. No significant difference of trunk neural crest cell migration could be detected between wild-type and draxin knockout mice. This result might be caused by the redundant function of multiple genes in ovo.
